# Initial experience with transcatheter aortic valve replacement before and after lung transplant

**DOI:** 10.1016/j.xjse.2024.100022

**Published:** 2024-09-07

**Authors:** Mujtaba Mubashir, John O. Barron, Samir Kapadia, Amar Krishnaswamy, Shinya Unai, Kenneth McCurry, Marie Budev, Grant Reed, Rishi Puri, Haytham Elgharably, Usman Ahmad, James Yun

**Affiliations:** a Department of Thoracic and Cardiovascular Surgery, Cleveland Clinic, Cleveland, Ohio; b Department of Cardiology, Cleveland Clinic, Cleveland, Ohio; c Department of Pulmonary and Critical Care Medicine, Cleveland Clinic, Cleveland, Ohio

**Keywords:** TAVR, aortic valve, transcatheter aortic valve, lung transplant

## Abstract

**Objectives:**

Patients with end-stage lung disease and lung transplant (LTx) recipients are high-risk candidates for surgical aortic valve replacement (AVR). Transcatheter AVR (TAVR) is an established treatment for aortic stenosis. Experience with TAVR in the LTx population is limited. We sought to report our experience in patients who underwent TAVR before or after LTx.

**Methods:**

Single-center, retrospective analysis, including all patients who underwent TAVR pre- or post-LTx from 2000 to 2020. Indications, mode of anesthesia, timing of TAVR, and procedural outcomes were reviewed.

**Results:**

In total, 10 LTx patients underwent TAVR. Aortic stenosis was the indication for TAVR in all patients: 5 had TAVR pre-LTx, and 5 post-LTx. All 10 TAVRs were performed with transfemoral access, and 9 out of 10 with intravenous sedation and monitored anesthesia care; only 1 required general anesthesia. In the TAVR pre-LTx group, mean age was 62 years and 4 patients were men. Four TAVR valves were balloon-expandable and 1 self-expanding. Median time from TAVR to transplant was 7.4 months (range, 1.7-36 months), and median length of stay after TAVR 4 days (range, 2-36 days). The only TAVR-related complication was a pacemaker for heart block (1 out of 5). All 5 subsequently underwent successful double LTx (1 with concurrent liver transplant). In the TAVR post-LTx group, mean age was 66 years and 2 patients were men. Four TAVR valves were balloon-expandable and 1 was self-expanding. Median time from LTx to TAVR was 5 years (range, 2.6-7.6 years), and median length of stay post-TAVR was 2 days (range, 1-17 days). The only TAVR-related complication in this group was heart block requiring pacemaker (1 out of 5). In both groups, there were no intraprocedural mortalities or conversions to surgical AVR. No patients required post-TAVR surgical intervention during study follow-up.

**Conclusions:**

Our initial experience suggests that TAVR is safe and feasible for treatment of aortic stenosis in patients with end-stage lung disease awaiting LTx, and in patients who have undergone LTx. Overall TAVR-related complications included need for pacemaker. TAVR should be considered for treatment of aortic stenosis in patients with end-stage lung disease awaiting transplant, and for post-LTx patients as an alternative to surgical aortic valve replacement.

**Figure:**
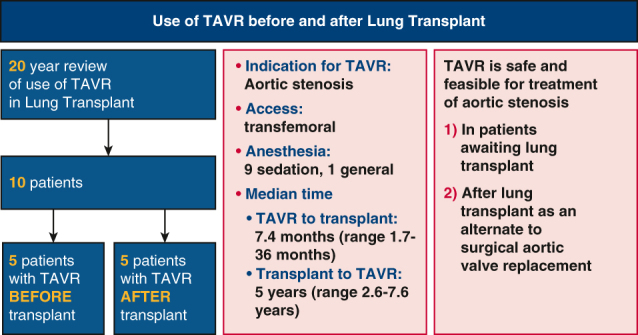
Safety of TAVR before and after lung transplant.

**Central Message**

TAVR is safe and feasible for treatment of aortic stenosis in patients with end-stage lung disease awaiting lung transplant and in patients who have undergone lung transplant previously.

**Perspective**

Currently, there is paucity of experience with regards to the long-term outcomes and safety profile of TAVR in the transplant population. Our case series is the largest to date that demonstrates the safety of TAVR, both in patients who have already undergone lung transplant, as well as in patients who are awaiting lung transplant.

The number of lung transplants (LTx) performed worldwide has increased to more than 4000 annually, more than double the volume in 2005.^1^ Despite this, 1- and 3-year survival after LTx are 80% and 65%, respectively, and remain lower than for other solid organ transplants.^1^^,^^2^ The current lung allocation score prioritizes acutely ill patients, many of whom are older with primary diagnosis of idiopathic pulmonary fibrosis (IPF).^3^ Therefore, aortic stenosis or other cardiac pathologies may be encountered and require treatment.^4^ Concomitant cardiac surgery, including aortic valve replacement (AVR), coronary artery bypass grafting, and closure of patent foremen ovale or atrial septal defect, can be performed at the time of transplant in selected patients, with good clinical outcomes.^5^, ^6^, ^7^, ^8^ However, if correction of aortic stenosis or cardiac disease is required before or after LTx, the presence of end-stage lung disease (ESLD) or the immunosuppressed posttransplant state increase surgical risk.

Since the first transcatheter aortic valve replacement (TAVR) in 2002,^9^ TAVR has rapidly gained acceptance for the treatment of aortic stenosis in not only high-risk surgical candidates,^10^ but also intermediate and low-risk surgical candidates.^11^^,^^12^ Currently, TAVR is feasible in >95% of patients with a transfemoral approach and conscious sedation only.^13^ Outcomes data for TAVR in the LTx population is very limited, with only a few case reports in the literature. Wallen and colleagues^14^ described 2 patients who successfully underwent TAVR as a bridge to LTx. Despite the routine use of TAVR in patients at high surgical risk, the outcomes of TAVR in the LTx population have not been defined. We sought to report our initial experience with patients who underwent TAVR either before or after LTx.

## Patients and Methods

A retrospective review of all patients at the Cleveland Clinic who underwent TAVR either before or after LTx between 2007 and 2020 was performed. The electronic medical record was queried to include all patients who underwent TAVR as well as any LTx during this period. A total of 10 patients who satisfied both criteria were identified, as part of a multisurgeon experience. The LTxs for these 10 patients were performed 2009 to 2020, whereas TAVRs took place between 2014 and 2020. Each case was reviewed with patient demographics, indications for transplant and TAVR, postprocedural details, and outcomes for both TAVR and transplant analyzed. Statistical analysis included simple descriptive statistics to report mean, median, and ranges for quantitative variables using SPSS version 22 (IBM-SPSS Corp). A description of each patient was reported in a tabular form. This study was approved by the institutional review board at our institution (#19-140; approved February 8, 2019).

## Results

### Overall Results

A total of 10 LTx patients underwent TAVR for aortic stenosis; of these, 5 had TAVR pre-LTx (Table 1), and 5 patients underwent TAVR post-LTx (Table 2). All TAVRs were performed using transfemoral access. Of the 10 TAVRs, 9 were performed with intravenous sedation and monitored anesthesia care (MAC). Only 1 patient required general anesthesia due to severity of her lung disease (Patient D). The majority of patients underwent double LTx (8 out of 10) versus single LTx (2 out of 10). Transplant indications were: IPF (7 out of 10), severe bronchiectasis (1 out of 10), alpha-1 antitrypsin deficiency (1 out of 10), and usual interstitial pneumonia (1 out of 10). Mean age at transplant was 64 years, and 6 were men. 1 patient in each group required a permanent pacemaker (patient A and H), although mortality in both these patients were LTx-related. In both groups, there was no immediate procedural mortality associated with TAVR or LTx. Late mortalities were related to LTx-related issues. No TAVR valve explantation was performed in either group.

**Table 1:** Description of patients with transcatheter aortic valve replacement (TAVR) before lung transplant (LTx)

Patient	Age at LTx (y)	LTx type	LTx indication	Time from TAVR to LTx (mo)	TAVR indications and echo peak/mean gradient (mm Hg)	TAVR valve	LOS after TAVR (d)	TAVR complications	Pretransplant echo peak/mean gradient (mm Hg)	Survival data∗
A	59	Double lung and liver	Alpha-1 antitrypsin deficiency/COPD	14.5	Moderate to severe AS 70/41	26 mm Edwards Sapien S3	4	Complete heart block requiring fixation lead placement complicated by cardiac tamponade, pericardial drain placement, and permanent pacemaker	No AR 25/11	Deceased 3.6 y post-TAVR (cardiogenic shock, severe AS, pneumonia)
B	68	Double lung	IPF/chronic hypersensitivity pneumonitis	2.5	Moderately severe AS 43/22	26 mm Edwards Sapien S3	36	Respiratory failure (exacerbation of hypersensitivity pneumonitis and worsened pulmonary HTN) expediting transplant listing	Mild AR 22/11	Deceased 7.3 y post TAVR (BOS)
C	62	Double lung	IPF	1.7	Moderately severe AS 55/29	26 mm Edwards Sapien S3	2	Stroke 2 mo after TAVR during post-LTx period (embolic protection device placed during TAVR)	Mild AR 28/13	Deceased 2.4 y post TAVR (LTx rejection, respiratory failure)
D	64	Double lung	Bronchiectasis/lupus/Sjogren syndrome	26	Severe AS 112/62	29 mm Corevalve Medtronic Evolut R valve	5	None	Mild AR 31/15	Deceased 3.2 y post TAVR (FTT, chronic hypercapnic and hypoxemic respiratory failure with superimposed pneumonia)
E	57	Double lung	IPF	7.4	Severe AS 96/63	26 mm Edwards Sapien S3	3	MRSE endocarditis 2.5 y after TAVR requiring lifelong antibiotics	Mild AR 29/15	Deceased 3.6 y post TAVR (septic shock in setting of infective endocarditis)

*Echo*, Echocardiograph; *LOS*, length of stay; *COPD*, chronic obstructive pulmonary disease; *AS*, aortic stenosis; *AR*, aortic regurgitation; *IPF*, idiopathic pulmonary fibrosis; *HTN*, hypertension; *BOS*, bronchiolitis obliterans syndrome; *FTT*, failure to thrive; *MRSE*, Methicillin-resistant Staphylococcus epidermidis.

∗ As of July 6, 2024.

**Table 2:** Description of patients with transcatheter aortic valve replacement (TAVR) after lung transplant (LTx)

Patient	Age at LTx (y)	LTx type	LTx indication	Time to TAVR (y)	TAVR indication and echo peak/mean gradient (mm Hg)	TAVR valve	LOS after TAVR (d)	TAVR complications	Post-TAVR echo peak/mean gradient (mm Hg) at 1 y	Survival data∗
F	66	Double lung	IPF (in setting of remote Agent Orange exposure)	5	Severe AS 50/28	29 mm Edwards Sapien S3	2	None	No AR –/15	Alive
G	61	Left single lung	Polymyositis (Jo-1 syndrome)/pulmonary fibrosis	4.7	Severe AS 67/34	23 mm Edwards Sapien XT	4	Valve in valve	Trivial AR 20/11	Deceased 2.8 y post TAVR (CLAD/BOS)
H	66	Right single lung	Interstitial lung disease-usual interstitial pneumonia	7.6	Severe AS 97/64	23 mm Edwards Sapien S3	17	Complete heart block requiring permanent pacemaker with RV perforation requiring median sternotomy - Stroke requiring ICA stent 1 y post-TAVR - Need for CFA balloon dilation post TAVR at access site	Trivial AR 36/21	Deceased 1.5 y post TAVR (aspiration pneumonia)
I	64	Double lung	IPF (asbestos exposure)	5.2	Moderately severe AS 61/36	26 mm Edwards Sapien S3	1	None	Trivial AR 21/12	Alive
J	76	Double lung	IPF	2.6	Moderate AS 35/21	23 mm Edwards Sapien S3	1	None	Mild AR 36/20	Deceased 2.8 y post TAVR (CMV viremia, mucormycosis, pneumonia)

*Echo*, Echocardiograph; *LOS*, length of stay; *IPF*, idiopathic pulmonary fibrosis; *AS*, aortic stenosis; *AR*, aortic regurgitation; *CLAD*, chronic lung allograft dysfunction; *BOS*, bronchiolitis obliterans syndrome; *RV*, right ventricle; *ICA*, internal carotid artery; *CFA*, common femoral artery; *CMV*, cytomegalovirus.

∗ As of July 6, 2024.

### TAVR Before LTx

In the TAVR pre-LTx group (Table 1), 4 TAVRs were performed using a balloon-expandable valve (Edwards Sapien S3) and 1 with a self-expanding valve (CoreValve Medtronic Evolut R valve). Mean age at TAVR was 62 years and 4 were men. Median time from TAVR to transplant was 7.4 months, with the shortest interval between TAVR and transplant 1.7 months (total range, 1.7-36 months). In 1 case, a permanent pacemaker was required after TAVR for complete heart block (patient A). Median length of hospital stay after TAVR was 4 days (range, 2-36 days). All 5 subsequently underwent successful double LTx (1 with concurrent liver transplant). Of note, there were no patients in our experience who were on the transplant list, and did not undergo transplantation after TAVR. One patient had a stroke 4 days after LTx (patient C). With regard to late mortality, all were related to various LTx related morbidities, including pneumonia, bronchiolitis obliterans, failure to thrive, and hypoxemic and hypercapnic respiratory failure. Of note, 1 patient was incidentally found to have severe aortic stenosis at time of mortality related to LTx complication (patient A). Another patient developed infective endocarditis ∼2.5 years after LTx (patient E), which was treated medically; the patient ultimately died >3.5 years posttransplant due to multisystem organ failure.

### TAVR After LTx

Of the 5 patients who underwent TAVR post-LTx (Table 2), 4 balloon-expandable (Edwards Sapien) and 1 self-expanding (Medtronic CoreValve Evolut R) valves were implanted. Mean age was 66 years and 2 were men. Median time from LTx to TAVR was 5 years (range, 2.6-7.6 years). Median length of stay after TAVR was 2 days (range, 1-17 days), with most patients discharged between postprocedure days 1 and 4. One patient had valve-in-valve TAVR for aortic insufficiency noted at placement of the first valve (patient G). Another patient (patient H) had a TAVR complicated by complete heart block requiring pacemaker complicated by tamponade requiring median sternotomy and right ventricular repair. The patient was discharged on post-TAVR day 17. With regard to late mortality, similar to the other group, all were related to various LTx-related morbidities, including pneumonia, bronchiolitis obliterans, and septic shock, as seen in Table 2.

## Discussion

### Current Role of TAVR in Patients Pre- and Post-LTx

The literature on use of TAVR before LTx is sparse. There are only a select few case reports describing this, which is expected given the technology is relatively new. Wallen and colleagues^14^ reported 2 cases of TAVR for aortic stenosis as a bridge to successful transplant. One patient was a 66-year-old man with IPF (diffusing capacity of the lungs for carbon monoxide, 27% predicted) who had severe aortic stenosis, with a transvalvular mean gradient of 39 mm Hg, and peak gradient of 63 mm Hg, After multidisciplinary committee evaluation, the patient underwent uncomplicated transfemoral TAVR with a 29 mm Sapien 3 valve, followed by uneventful double LTx 56 days later. A second patient in the same case series was a 70-year-old man with hypersensitivity pneumonitis, and severe bioprosthetic aortic stenosis after surgical aortic valve replacement (SAVR) 12 years earlier. This patient underwent successful transfemoral valve-in-valve TAVR with a 26-mm Evolut valve with general anesthesia; he underwent successful single right LTx 103 days later.^14^ Another study by Elbadawi and colleagues^15^ on the outcomes of TAVR versus SAVR among solid organ transplant recipients reported the use of valvular intervention in LTx; however, did not further characterize the type of intervention in these patients due to the small sample size in patients undergoing LTx versus other solid organ transplants encountered, including liver and kidney.^15^ With regard to type of LTx (double or single), 2 patients (G and H) had received single LTx before TAVR; however, this was unrelated to degree of aortic stenosis, and in our experience the degree of aortic stenosis does not influence type of LTx performed. Our study, to our knowledge, is the largest series to date describing successful outcomes of TAVR pre- and post-LTx.

### Modality of Anesthesia in TAVR

General anesthesia in patients with ESLD, including interstitial lung disease is associated with increased postoperative pulmonary complications compared with the general population, with increased risk of pneumonia, exacerbation of pulmonary insufficiency, increased anesthesia time, and increased length of hospital stay.^16^ LTx candidates by definition have limited pulmonary functional reserve, and thereby become high risk for cardiac surgery; they benefit from cardiac procedures in which general anesthesia can be avoided. Several large studies comparing TAVR performed under general anesthesia versus MAC have consistently demonstrated shorter procedural time and fluoroscopy time, less need for vasopressors and inotropes, briefer intensive care unit stay, and shorter hospital stay while at the same time showing no compromise in the safety or efficacy profile of the procedure.^13^^,^^17^ In our study, performance of TAVR using MAC in 4 out of 5 pre-LTx patients contributed ultimately to successful bridge to LTx. One patient underwent TAVR with general anesthesia (patient D). This patient was electively intubated and extubated at the end of the procedure. Intubation was performed for airway control. One year later, this patient had respiratory decompensation that resulted in listing for transplant.

Of the 5 patients who underwent TAVR before LTx, 2 patients (B and D) were not undergoing transplant evaluation of the time of TAVR. Patient B had increased oxygen requirements, which led to expedited evaluation for transplant listing during that hospitalization. Patient D developed respiratory insufficiency approximately 1 year after TAVR, which prompted evaluation and listing for transplant. All patients who underwent TAVR after LTx also had TAVR with MAC, without any notable periprocedural respiratory events at the time of TAVR.

Our initial experience thus suggests that patients with ESLD undergoing TAVR are still at some risk for respiratory decompensation even with avoidance of general anesthetic, and that careful assessment of pre-TAVR pulmonary function and reserve is necessary.

### TAVR as a Bridge to LTx

Recent large trials suggest that transfemoral TAVR is safe, and at the very least equivalent, if not superior, to SAVR in the treatment of low- and intermediate-risk surgical candidates.^11^^,^^18^ Patients with ESLD who require LTx are by definition high risk for SAVR. Although it is potentially possible to perform SAVR in lieu of TAVR for these patients, either before transplant listing or at the time of LTx, TAVR offers a fully percutaneous treatment option that avoids cardiopulmonary bypass. In practice, the outcomes of our series of patients, taken together with previous reports such as by Wallen and colleagues,^14^ provides further evidence that it is reasonable to consider TAVR to treat aortic stenosis in selected patients that are (or soon will be) LTx candidates. Further data are needed to better define the role of TAVR in patients in need of LTx.

### Role of Reinterventional Cardiac Surgery After TAVR in Patients Undergoing LTx

Yun and colleagues^19^ showed that in recent years, more cardiac surgery is being performed after TAVR procedures in nontransplant patients. From 2012 to 2018, fewer than 10 operations were performed after TAVR, but 18 were performed in 2019 at the Cleveland Clinic and these interventions were performed much sooner, usually around the 1-year mark.^19^ TAVR valve stenosis/regurgitation was the leading indication, followed by paravalvular leak in and endocarditis. This raises an important question in the transplant population. If patients awaiting LTx develop complications from TAVR, they may require complex cardiac surgery either before LTx or at the time of LTx. Although concomitant cardiac surgery has been described in earlier studies,^2^^,^^8^ no studies describe this sort of surgery in the setting of a prior TAVR, which would considerably add to the complexity of transplant and perioperative care. Moreover, if patients develop complications after TAVR, there is a chance that they may no longer be eligible for transplant. Given that the number of TAVRs performed are increasing such that they now comprise 12.5% of all AVRs,^20^ transplant physicians will be faced with an increasing number of these complex scenarios, and clear guidelines and protocols will have to be established.

### The Future of TAVR in LTx

Although TAVR continues to become widely adopted in high-risk patients, currently there are not enough data and experience about the long-term outcomes and safety profile of TAVR in the transplant population. Although it does appear to be safe and feasible in a small number of patients to date, further clinical experience is required, especially with regard to comparison of TAVR versus SAVR. This is a much-needed comparison, which due to overall low case volumes would likely require pooled cases from several institutions to be able to make a valid comparison. Regardless, given the growth in both LTx and TAVR procedures over time, the use of TAVR in LTx candidates and recipients is likely to increase with time and may prove to be a viable option for patients with severe aortic stenosis.

## Conclusions

Our initial experience suggests that TAVR is safe and feasible in patients with ESLD awaiting LTx, and in patients who have previously undergone LTx. The primary TAVR complication observed was need for pacemaker. TAVR may be considered for patients with ESLD awaiting transplant and in patients post-LTx as an alternative to SAVR, although additional studies are needed.

## Conflict of Interest Statement

The authors reported no conflicts of interest.

The *Journal* policy requires editors and reviewers to disclose conflicts of interest and to decline handling or reviewing manuscripts for which they may have a conflict of interest. The editors and reviewers of this article have no conflicts of interest.
